# An investigation of neuromelanin distribution in substantia nigra and locus coeruleus in patients with Parkinson’s disease using neuromelanin-sensitive MRI

**DOI:** 10.1186/s12883-023-03350-z

**Published:** 2023-08-14

**Authors:** Qiang Liu, Pan Wang, Chenghe Liu, Feng Xue, Qian Wang, Yuqing Chen, Ruihua Hou, Teng Chen

**Affiliations:** 1 Department of Neurosurgery, Qilu Hospital of Shandong University, Jinan City, Shandong Province China; 2 Department of Radiology, Qilu Hospital of Shandong University, Jinan City, Shandong Province China; 3 School of Clinical Medicine Addenbrooke’s Hospital, University of Cambridge, Cambridge, UK; 4 Clinical and Experimental Sciences, Faculty of Medicine, University of Southampton, Southampton, UK

**Keywords:** Parkinson’s Disease, Neuromelanin, Neuromelanin-sensitive MRI, Substantia Nigra, Locus Coeruleus

## Abstract

Loss of neuromelanin in the midbrain is known in Parkinson’s disease(PD), which can now be directly detected by neuromelanin-sensitive MRI(NM-MRI). This case-control study was to investigate the distribution of neuromelanin in the substantia nigra(SN) and the locus coeruleus(LC) using NM-MRI technique and evaluate its potential as a diagnostic marker for PD. 10 early PD patients(H&Y stage I, II), 11 progressive PD patients(H&Y stage III-V), and 10 healthy controls matched in age and gender were recruited. All participants completed clinical and psychometric assessments as well as NM-MRI scans. Neuromelanin signal intensities in SN and LC were measured by contrast-to-noise ratios(CNRs) derived from NM-MRI scans. There were significant decreases of CNRs in SNpc(including anterior, central, and posterior) and LC in PD patients compared to controls. There were also significant differences of CNR between the left and right sides. CNR in LC had a negative correlation with the Non-Motor Symptoms Scale(NMSS) score in PD patients(|R|=0.49), whereas CNR in SNpc did not correlate with Unified Parkinson Disease Rating Scale(UPDRS) score(|R|<0.3). The receiver operating characteristic(ROC) curves revealed that the CNR in LC had a high diagnostic specificity of 90.1% in progressive patients. This study provides new evidence for the asymmetric distribution of neuromelanin in SN and the LC of patients with PD. The neuromelanin loss is bilateral and more predominately in LC than that in SN. This distinct neuromelanin distribution pattern may offer a potential diagnostic marker and a potential neuropharmacological intervention target for PD patients.

## Introduction

Parkinson’s disease(PD) is a common neurodegenerative disease. As the global aging population accelerates, the global incidence of PD is expected to double from approximately 7 million in 2015 to approximately 14 million in 2040 [1]. The understanding of the aetiology and development mechanisms still remains diverse and unclear [2]. At present, its clinical diagnosis still mainly relies on traditional symptomatic assessment and the response to levodopa replacement therapy which have limitations [3]. Therefore, there has been a pressing need for searching for new diagnostic markers and novel treatment targets for PD in recent years.

Neuromelanin is an electron-dense brown/black pigment from the formation of dopamine, norepinephrine or other catecholamines in the cytoplasm oxidized by tyrosinase. It is a downstream product of levodopa’s oxidation in the brain [4]. Earlier molecular histological studies demonstrate that neuromelanin is widely distributed within dopamine neurons of substantia nigra pars compacta(SNpc) in midbrain and within noradrenergic neurons of locus coeruleus(LC) in pons [5–7]. It is known that the mechanism of motor symptoms(such as static tremor, rigidity, bradykinesia and postural instability) of PD is due to the degeneration and apoptosis of dopamine neurons in the SNpc, resulting in the imbalance of dopamine-acetylcholine pathway between substantia nigra and striatu. In addition, all PD patients also experience a series of non-motor symptoms during their disease progression such as problems with wakefulness and sleep, cognitive changes, anxiety, depression, and psychotic symptoms, in particular in the early phase of disease [17]. More research is needed to investigate the mechanisms underlying these non-motor symptoms and one important nucleus deserves attention is the locus coeruleus(LC), which is a bilateral nucleus located in the dorsal pontine tegmentum(PT). It is the major source of noradrenaline(NA), a neuromodulator that plays a key in cognition [2] [8]. Evidence so far have indicated that both the SNpc and the LC experience apoptosis of neurons in the development of PD, in other words, accompanied by the loss of neuromelanin. During the synthesis of neuromelanin, catecholamine oxidative metabolism requires the participation of metal ions such as iron, copper, and zinc, and the resulting melanin particles also form chelates with these ions and are stored in lysosomes together [9, 10]. This makes neuromelanin paramagnetic in a magnetic field, which makes it possible to be detected directly using magnetic resonance imaging(MRI).

The paramagnetic effect makes longitudinal relaxation time of neuromelanin in the magnetic field much shorter, thus forming a high signal echo on T1-weight, which is called neuromelanin-sensitive MRI(NM-MRI) [11]. As early as the beginning of this century, *Sasaki and Otsuka, et al.* [12–14], a group of Japanese scholars, took the lead in using the technology in PD patients, and their preliminary data demonstrated positive correlations between the signal intensity of SNpc and LC and neuromelanin concentration, indicating the potential use of NM-MRI in the diagnosis of PD. This has led to growing research interest in exploring the use of NM-MRI in neurodegenerative diseases in recent years. *Okuzumi and Hatano, et al.* [15] used NM-MRI and DaT-SPECT to quantify the signal intensity of SN in PD patients with motor fluctuations and abnormal involuntary movements(AIMs), suugesting that NM-MRI has a better advantage than the latter on distinguishing the progression of advanced PD. Moreover, *Takahashi, et al.* [16] conducted extensive work investigating the changes of neuromelanin signal in SNpc of early PD patients combining quantitative susceptibility mapping(QSM) with NM-MRI and suggested that NM-MRI can be an important tool for early diagnosis of PD. This exploratory study was to further investigate neuromelanin distribution in the SN and the LC in patients with PD using NM-sensitive MRI in a case-control study. We hypothesised that there may be different patterns of neuromelanin distributions in the SN and the LC in patients with PD at different stages comparing to health controls.

## Methods

### Study design

A cross-sectional case-control study design was employed. All potential participants were screened, and PD diagnoses were validated. All eligible participants gave their written informed consent before taking part in the study. The research protocol was approved by the Research Ethics Committee of Shandong University Qilu Hospital. The study was conducted from June 2021 to June 2022.

### Participants

21 patients who met the International Parkinson and Movement Disorder Society(MDS) Clinical Diagnostic Criteria for idiopathic PD including 10 early patients(I, II) and 11 progressive patients(III-V) according to Hoehn-Yahr stage were recruited from the outpatient clinic and wards of neurology department and neurosurgery department at Shandong University Qilu Hospital. 10 Healthy subjects matched in sex and age were recruited from the local community. All participants were drug-naïve. Participants with any other medical conditions or taking any medication which might potentially affect the LC noradrenergic pathway were excluded. Current use of medications with an NA-based mechanism of action refers to selective noradrenaline reuptake inhibitors such as atomoxetine, maprotiline, reboxetine, and viloxazine, and α2-adrenoceptor antagonists such as atipamezole, efaroxan, idazoxan, and yohimbine. All participants were right-handed.

### Neuropsychometric measures

Mini-mental state examination(MMSE) and Montreal cognitive assessment(MoCA) were used to evaluate the degree of cognitive impairment. Depression and anxiety symptoms were examined by Hamilton depression scale(HAMD) and Hamilton anxiety scale(HAMA). Severity of motor and non-motor symptoms were quantified by Part III of the Unified Parkinson’s Disease Rating Scale(UPDRS) and Hoehn-Yahr(H&Y) stage as well as Non-Motor Symptom Assessment Scale(NMSS). The scoring of all scales were double checked by two clinical researchers.

### Magnetic resonance imaging protocol

**Fig. 1:**
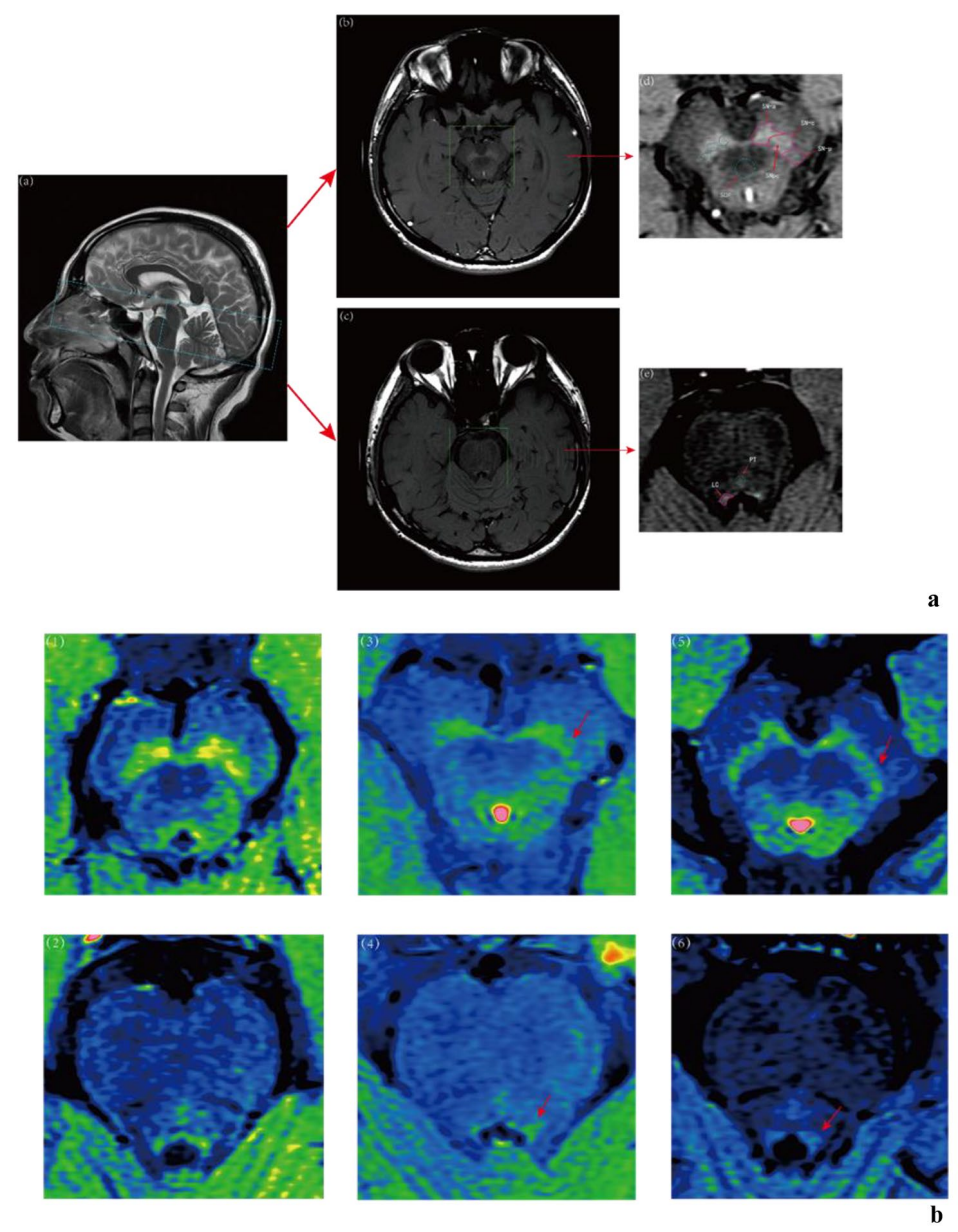
**1a**. Imaging processing protocol of NM-MRI. **(a)** showed a T2-weighted sagittal view of a healthy 40-year-old man. And the blue frame was the initial scanning location and vertical to the bottom of fourth ventricle, ranging from splenium of the corpus callosum to the inferior edge of pons. The high signal areas of **(b)**,**(d)** and **(c)**,**(e)** was the coronal images of substantia nigra pars compacta (SNpc) and locus coeruleus (LC) on NM-MRI of a healthy 23-year-old man. The blue circles were the measurement ROIs.SN-a, SN-c, SN-p and SCP in **(d)** indicate anterior part of SNpc, central part of SNpc, posterior part of SNpc and superior cerebellar peduncle. PT in **(e)** indicates pontine tegment (PT). **1b** Sample images of comparison of CNR of SNpc and LC between groups (1),(2) were SNpc and LC of a healthy 40-year-old male. (3),(4) were SNpc and LC of a 49-year-old male with early PD(H&Y stage:II). (5),(6) were SNpc and LC of a 64-year-old female with progressive PD(H&Y stage:IV)

MRI data were acquired by a 3.0T magnetic resonance equipment(Discovery ^TM^ MR750, GE Healthcare, Milwaukee, WI, USA). Scan parameters of neuromelanin-sensitive T1-weighted imaging were as follows: Fast spin-echo; Echo trains:2;TE/TR:600/14 ms; Slice thickness:3.0 mm with no gap; Number of slices:12; Field of view(FOV):24.0 cm; Matrix size: 512 × 320; NEX: 5; Acquisition time: 9 min 44 s [18]. The initial scanning frame was located on the T2-weighted sagittal image, ranging from splenium of the corpus callosum to the inferior edge of pons and being vertical to the bottom of fourth ventricle. At the same time, the conventional axial T1 and T2-weighted sequences, as well as FLAIR and DWI were also collected to exclude any coexisting central nervous system diseases that would interfere signal measurement such as stroke. The imaging processing protocol was illustrated in Fig. 1.

### Imaging data processing

All NM-MRI signal data were measured using the Advantage Workstation(AW4.7 ext.16). All data were backed up by DVD dishes for the convenience of measurement, and the further image processing was completed with DICOM Image Viewer supported by GE Healthcare. A series of NM-MRI consisted of twelve consecutive slices containing three of SNpc and two of LC. For SNpc, the best observing and measuring level was decussation of superior cerebellar peduncle(SCP), while pontine tegment(PT) plane was selected for LC as far as possible. Considering individual structural differences contrast-to-noise ratio(CNR) were calculated for comparison. The premise was that the signal intensity of nerve conduction tract maintained stable on MRI in PD patients. Then we selected decussation of superior cerebellar peduncle(Region Of Interest: 20mm^2^) as the reference area of SNpc, and selected pontine tegment area (Region Of Interest: 5mm^2^) as the reference area of LC (Region Of Interest: 1mm^2^) [14]. Because of SNpc’s larger size, we divided the nucleus mass into three sections: anterior part of SNpc(SN-a), posterior part of SNpc(SN-p) and the central part of SNpc(SN-c) [18], to measure signal numerical values respectively (Region Of Interest: 5mm^2^), see Fig. 1.

All measurements were performed and collected by a consultant radiologist, and the second radiologist remeasured signal numerical values to double check the imaging data collected by the first radiologist. Two radiologists were blind to participants information and worked independently. Each region of interest was obtained manually for three times and the mean value was calculated and used for analysis. The left and right nuclei were measured at the same time. The corresponding CNR value was calculated using the following formula:(Sig _Target area_-Sig _Reference area_)/ (Sig _Reference area_) [14]. CNR of each nucleus section was calculated by averaging the left and right sides.

### Statistical analysis

Statistical analyses were conducted using R software (Version R4.1.3 for Windows https://mirrors.pku.edu.cn/CRAN/). Independent Mann-Whitney U-test and Chi-squared test were conducted to compare demographic and clinical characteristic differences between groups. Independent t-test was used to examine differences of CNR between groups and differences of CNR between SNpc and LC, including left and right side, as well as anterior, central, and posterior site. Analysis of variance (ANOVA) was conducted to investigate the changes of CNR in SNpc and LC between groups. Multivariate correlation analyses were conducted to check whether clinical features were independently correlated to CNR of SNpc and LC. In addition, receiver operator characteristic (ROC) curves were drawn to explore the sensitivity and specificity of using NM-MR in diagnosing PD. Internal Consistency Coefficient (ICC) was also calculated to assess the consistency of measurements collected by two independent radiologists. All above tests were chosen as appropriate depending on the type of data distribution. All statistical tests were two-tailed, and the significance level was set to 0.05 (P < 0.05).

## Results

### Demographic and clinical characteristics of PD patients and controls

Demographic and clinical characteristics data were presented in Table 1. As expected, duration of disease (P = 0.003) and UPDRS Part III motor scores (P = 0.05) of progressive group were significantly higher than those of early-stage group, whereas there was no significant difference in the measures of NMSS MMSE, MoCA, HAMD and HAMA between the early stage and progressive patients. When compared with healthy subjects, there were significant differences in scores of NMSS, MMSE, and MoCA in both early and progressive group (P < 0.05). However, there was only significant difference between progressive patients and healthy controls in HAMD (P < 0.05) not in HAMA (P > 0.05). It should be noted that both early and progressive PD group had mild cognitive impairment based on MOCA assessment.

**Table 1:** Demographic and clinical characteristics of PD patients and controls

		PD			Healthy Subjects N = 10	P value^*^
		Early Patients N = 10	Progressing Patients N = 11	Total N = 21		
Age(years)	Range(Median ± SD)	37 - 63(52.8 ± 8.18)	49 - 78(62 ± 11.09)	37 - 78(57.61 ± 10.67)	23 - 71(55 ± 15.59)	p_1_ = 0.11 p_2_ = 0.38 p_3_ = 0.88
Gender(male or female)	Males/Females	2/8	9/2	11/10	7/3	p_1_ = 0.017 p_2_ = 0.07 p_3_ = 0.59
Duration of disease	Range(Median ± SD)	1 - 7(4.6 ± 1.56)	5 - 12(7.55 ± 2.25)	1 - 12(6.17 ± 2.42)	-	p_1_ = 0.003 p_2_ < 0.05 p_3_ < 0.05
Hoehn-Yahr stage	Range(Median)	I-II(1.8)	III-IV(3.27)	I-IV(2.57)	-	p_1_ < 0.05 p_2_ < 0.05 p_3_ < 0.05
UPDRS Part III	Range(Median ± SD)	9 - 43(29 ± 12.28)	29 - 62(45.1 ± 10.89)	9 - 62(37.86 ± 14.21)	-	p_1_ = 0.05 p_2_ < 0.05 p_3_ < 0.05
Side at onset	Right/Left	5/5	7/4	12/9	-	p_1_ = 0.85 p_2_ < 0.05 p_3_ < 0.05
Motor fluctuation^#^ end of dose deterioration on-off phenomenon	Appearance(Yes or No)	Yes No	Yes Yes	- -	- -	- -
NMSS	Range(Median ± SD)	2 - 104(39.6 ± 29.90)	9 - 147(51.18 ± 36.48)	2 - 147(45.67 ± 33.21)	-	p_1_ = 0.29 p_2_ < 0.05 p_3_ < 0.05
MMSE MoCA	Range(Median ± SD)	19 - 29(26 ± 3.21) 11 - 28(23.38 ± 5.32)	17 - 30(24 ± 4.61) 8 - 29(20.5 ± 7.65)	17 - 30(24.94 ± 4.02) 8 - 29(21.94 ± 6.54)	27 - 30(28.6 ± 1.37) 26 - 29(27.6 ± 1.35)	p_1_ = 0.44 p_2_ = 0.02 p_3_ = 0.007 p_1_ = 0.67 p_2_ = 0.01 p_3_ = 0.003
HAMD HAMA	Range(Median ± SD)	3 - 18(9.13 ± 6.49) 3 - 23(11.63 ± 6.49)	3 - 20(9.44 ± 4.88) 4 - 25(11.22 ± 6.53)	3 - 20(9.29 ± 5.51) 3 - 25(11.41 ± 6.97)	1 - 7(4.8 ± 2.25) 1 - 12(7.6 ± 3.75)	p_1_ = 0.63 p_2_ = 0.28 p_3_ = 0.03 p_1_ = 0.92 p_2_ = 0.48 p_3_ = 0.27

*:Mann–Whitney U-test or Chi-squared test

p1: Early Patients versus Progressive Patients; p2: Early Patients versus Healthy Subjects; p3: Progressive Patients versus Healthy Subjects

UPDRS Part III: Part III of Unified Parkinson’s Disease Rating Scale; NMSS: non-motor symptom assessment scale for Parkinson’s disease; MMSE: Mini-mental State Examination;MoCA: Montreal Cognitive Assessment; HAMD: Hamilton Depression Scale; HAMA: Hamilton Anxiety Scale

^#^: Whether there was occurrence of motor fluctuation in previous medication use or not

### Comparison of NM-MRI measures between groups

**Fig. 2:**
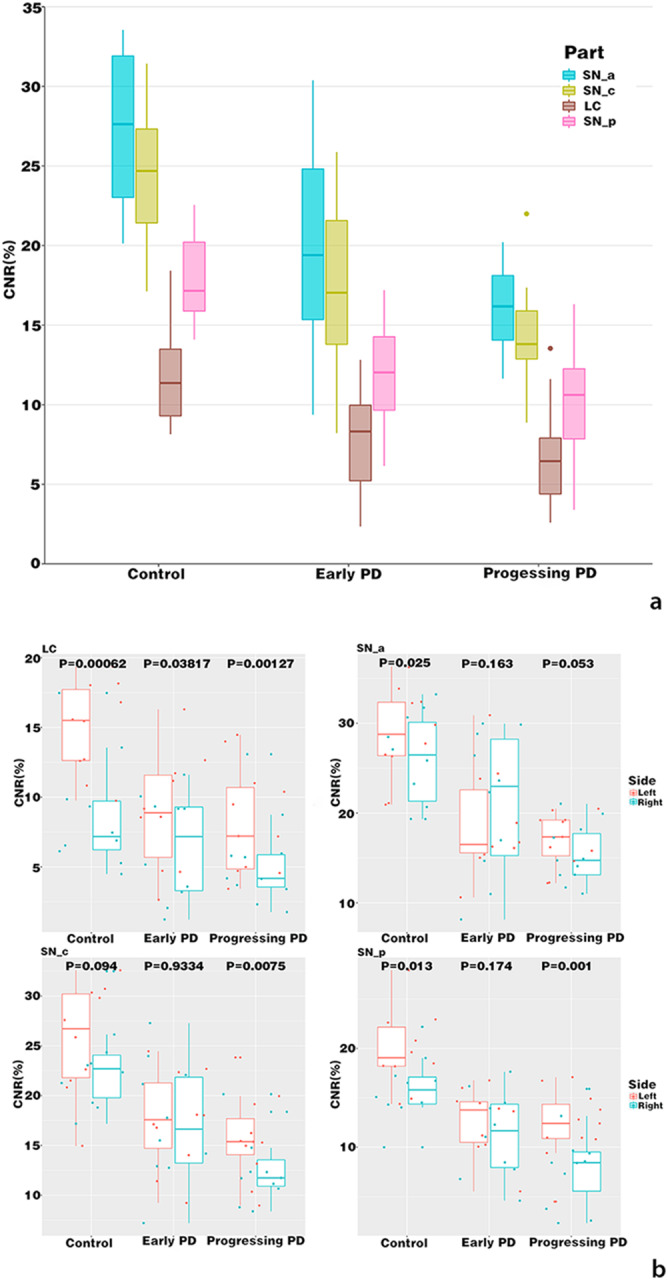
**2a** Comparison of CNR of SNpc and LC between groups. The CNR of each part of the SN and the LC decreased successively in healthy controls, early PD patients, and progressive PD patients (p < 0.05). **2b** Comparisons of CNR of bilateral SNpc and LC between groups. The CNR of the left side (dominant side) of the LC was significantly higher than that of the right side of the LC (p < 0.05), whereas this was only shown in the SNpc of healthy subjects and progressive PD patients, but not in early PD patients

Using the Advantage Workstation to render the colour levels of NM-MRI images, we could visually see a decrease of NM signal intensity both in SN and LC in a health volunteer, an early PD patient, and a progressive PD patient, see Fig. 1. In addition, ANOVA was used to analyse the differences between groups and post-hoc *t*-test was conducted to compare each two groups. It was found that there were significant difference between groups and the CNR levels of each part of the SN and the LC decreased successively in healthy people, early patients, and progressive patients (p < 0.05), and the CNR level of the SNpc showed a decreasing trend from the ventral side to the dorsal side, see Table 2; Fig. 2. It should be noted that the CNR of the left side (dominant side) of the LC was significantly higher than the right side of the LC (p < 0.05). However, this was only shown in the SNpc of healthy controls and progressive PD patients, but not notable in early PD patients, see Fig. 2.

**Table 2:** Comparison of CNR of SNpc and LC between groups

Part	Healthy Subjects(N = 10)	Early PD(N = 10)	Progressing PD(N = 11)	P value
SNpc				
anterior central posterior	20.14 ~ 33.55% (27.34% ± 4.90%) 17.12 ~ 31.43% (24.25% ± 4.38%) 14.10 ~ 22.55% (17.88% ± 3.08%)	9.37 ~ 30.38% (20.00% ± 6.60%) 8.21 ~ 25.87% (17.45% ± 5.42%) 6.15 ~ 17.19% (11.98% ± 3.61%)	11.64 ~ 20.20% (16.17% ± 2.92%) 8.88 ~ 21.99% (14.23% ± 3.67%) 3.40 ~ 16.31% (10.17% ± 3.59%)	P^*^<0.05 P_1_ = 0.011 P_2_ < 0.01 P_3_ = 0.021 P^*^<0.05 P_1_ = 0.006 P_2_ < 0.01 P_3_ = 0.028 P^*^<0.05 P_1_ = 0.001 P_2_ < 0.01 P_3_ = 0.043
LC	8.14 ~ 18.42% (11.81% ± 3.16%)	2.35 ~ 12.82% (7.74% ± 3.63%)	2.60 ~ 13.54% (6.83% ± 3.36%)	P^*^<0.05 P_1_ = 0.015 P_2_ < 0.01 P_3_ = 0.051

P*: Analysis of variance (ANOVA); P_1_: Healthy Subjects versus Early PD with t-test; P_2_: Healthy Subjects versus Progressing PD with t-test; P_3_: Early PD versus Progressing PD with t-test

### Associations between CNR and clinical characteristics

**Fig. 3:**
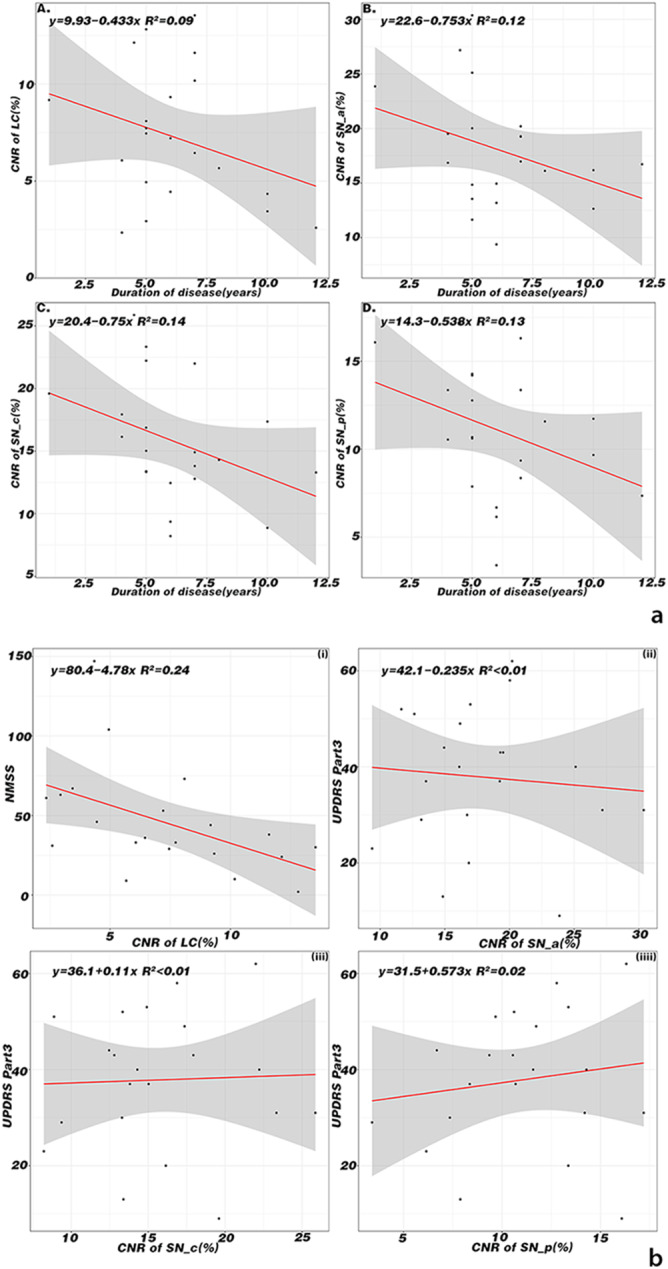
**3a** Correlation analysis between duration of disease and CNR of LC and SN in PD patients. Negative correlations between CNR of SN and LC and disease duration were found after multivariate linear correlation analysis. **3b** Associations between CNR of LC and NMSS and associations between CNR of each part of SN and UPDRS in PD patients. CNR of LC had a negative correlation with NMSS score of PD patients, while CNR of different part of SN was not correlated with UPDRS part III scores

Associations between CNR and clinical characteristics were also examined in the current study. Data analysis revealed a weak negative correlation between CNR of SNpc and LC and disease duration (|R|≥0.3) after multivariate linear correlation analysis(see Fig. 3). In addition, a linear fit model was used to examine correlations between NMSS scores and UPDRS part III scores with CNR of LC and SNpc, respectively. We found that CNR of LC had a negative correlation with NMSS scores of PD patients(|R| =0.49), while CNR of SNpc was not correlated with UPDRS part III scores(|R|<0.3), see Fig. 3.

### Specificity and sensitivity of using CNR of SN and LC in PD via NM-MRI

**Fig. 4:**
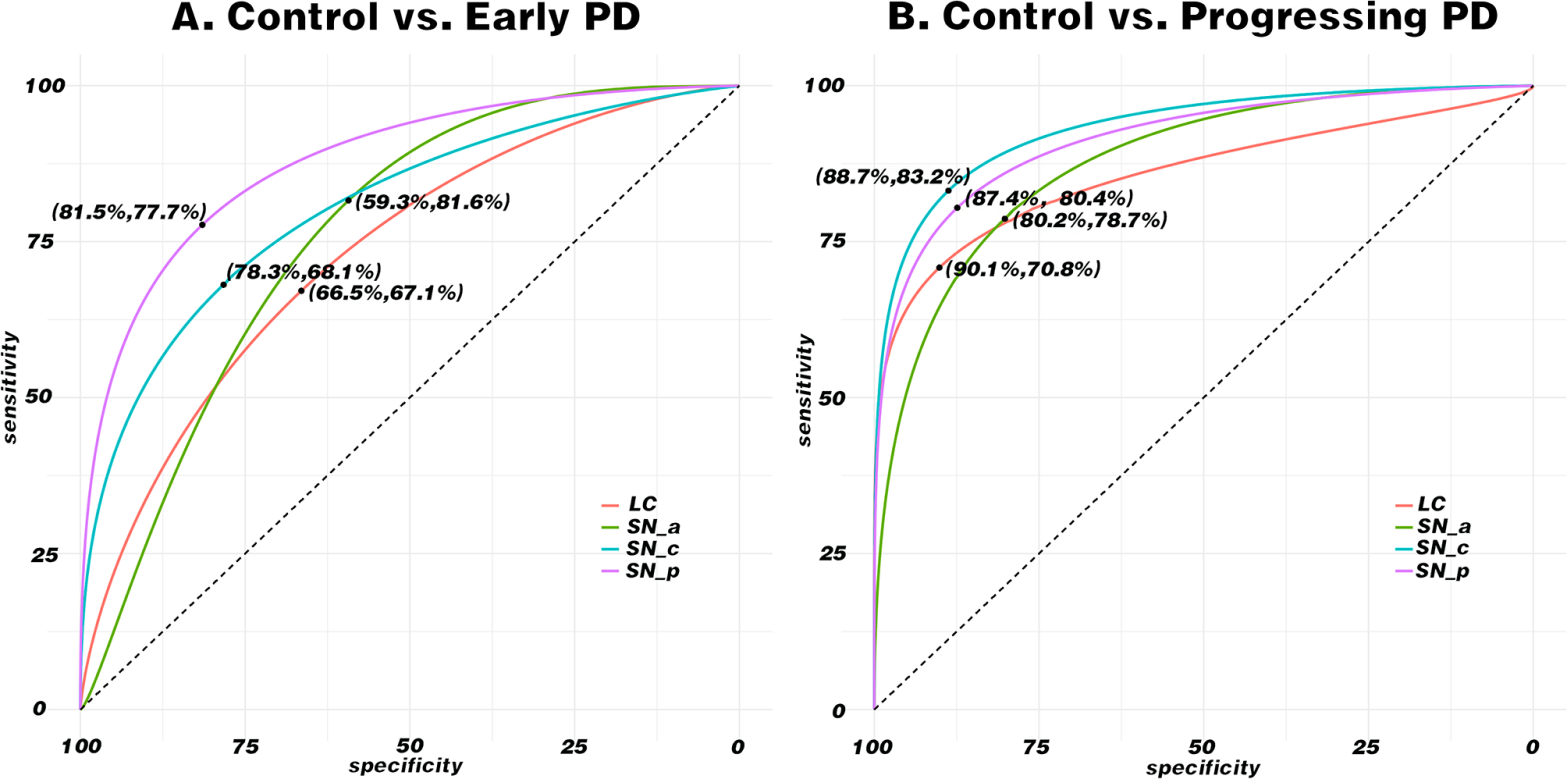
Receiver operating characteristic curves of different sources in SN and LC. **A:** the diagnostic value of CNR using NM-MRI in early PD patients. **B:** the diagnostic value of CNR using NM-MRI in progressive PD patients

The ROC curves were drawn to examine the specificity and the sensitivity of using CNR of SN and LC from NM-MRI images to diagnose PD were SN_a (59.3%, 81.6%), SN_c (78.3%, 68.1%), SN_p (81.5%, 77.7%) and LC (66.5%, 67.1%) in early patients, respectively. Findings from the ROC in progressive patients were SN_a (80.2%, 78.7%), SN_c (88.7%, 83.2%), SN_p (87.4%, 80.4%) and LC (90.1%, 70.8%). These findings indicate that CNR of SN showed higher specificity and sensitivity in progressive patients than that in early patients whereas the LC showed the highest specificity in progressive patients (90.1%), see Fig. 4. All cut-off values were based on that the Jordan index reached the maximum.

## Discussion

The current study successfully adopted a NM-MRI imaging technique to investigate neuronal loss by directly measuring neuromelanin distribution in both SN and LC in PD patients and matched healthy controls. The study was able to employ an optimised approach to detect small signal intensity changes in the early stage of PD by combining quantitative susceptibility mapping (QSM) analysis used by Takahashi and colleagues [16], as well as applying colour scale contrast to render NM-MRI images recommended by Sasaki et al. [19] and adjust the appropriate window width and window level to achieve optimal results. The study found significant associations between decreasing NM signal intensity in SNpc and LC, and the occurrence and development of PD, which is in line with previous research [12, 14, 18–20]. This case-control study provides new evidence of the asymmetric distribution of neuromelanin loss in bilateral SN and LC in patients with PD. The study also found that the neuromelanin loss was more predominant in the LC than that in the SN in early PD patients. This NM distribution pattern may offer a potential diagnostic marker for PD. Findings form this study may have important implications for future work into early diagnostic markers, predictors for treatment response, and novel intervention targets.

The study examined neuromelanin signal at different sections of the SNpc including the anterior, central and posterior part, and found a decreasing tendency of neuromelanin signal intensity from ventral to dorsal side. At the same time, the distribution of neuromelanin in the SN and the LC is asymmetrical, and the signal on one side is often higher than that on the opposite side(the left side is dominant in this study, perhaps because most of the research population is right-handed), and the LC also maintained synchronization of asymmetries throughout disease loss. Interestingly, this variability was observed with similar results in previous studies [18, 21–24]. Therefore, we assume that this difference may exist objectively, rather than caused by signal inequalities due to high magnetic fields and multi-channel coils. In addition, when it comes to the left and right dominant sides, we quickly thought that when PD motor symptoms involve both limbs, they often develop in an “N” shape, and one side is more severe than the other, which may reveal possible associations among the “dominant side” of neuromelanin distribution with handedness and onset side in PD. However, this asymmetry was not observed in the SNpc of early-stage patients, but was seen in progressive-stage patients, indicating that the speed of neuromelanin loss in the SN nuclei on both sides is inconsistent in the early stage of PD. In other words, the apoptosis rate of dopaminergic neurons in the SN was not uniform on both sides in the early stage of PD. In addition, the results show that the CNR of the SN is not directly related to the UPDRS Part III motor symptom score, and some studies have also reached similar conclusions [25, 26]. This is obviously not consistent with the currently recognized etiological mechanism [2], that is, the motor symptoms of PD are caused by the depletion of the SN-striatum dopaminergic system.

For this contradiction, it is not difficult to understand as during the progression of PD, the brain has a variety of compensatory loop mechanisms for the apoptosis of dopaminergic neurons in the SN compacta to supplement the depletion of presynaptic dopamine to slow down this apoptosis [27], such as in the early stage of PD, as a neurotransmitter, NE can supplement DA to a certain extent [28]. This can also explain that the severity of motor symptoms is not directly determined by the loss of DA in the SN compacta but depends on the balance between the brain compensatory circuit and the loss of DA in the SN. If this compensatory effect could complement for the function of lost dopamine enough in early PD, then patients show mild prodromal symptoms [29]. Only in progressive stage when the loop mechanism is decompensated, patients start to experience severe motor symptoms. Correspondingly, the previous problem is easily solved, that is, in the early stage of the disease, the SN nuclei on the non-dominant side may lose slowly due to the existence of a compensatory loop mechanism, while the dominant side maintains a high loss rate, which in turn leads to the loss of neuromelanin on both left and right side. The apoptosis rate of dopamine neurons in the cytoplasmic nuclei is not uniform, and during the decompensation period, the asymmetry of neuromelanin distribution appear in both nuclei again. This also proves that the loss of neuromelanin in the SNpc and the LC is a continuous process, and other brain compensatory circuit mechanisms only play a supplementary role and affect the speed of loss but cannot reverse the loss process [30]. More research is needed to confirm specific underlying mechanisms.

The study also found negative correlations between CNR of LC and NMSS scores and the synchronous asymmetry loss of neuromelanin in bilateral nuclei, suggesting that the speed of apoptosis of noradrenergic neurons in LC was not affected by other compensatory mechanisms. This indicates that in the early stage of PD, when apoptosis occurs in NE neurons of the LC, patients experience a series of symptoms of norepinephrine system disorders, such as hyposmia, repaid eye movement sleep behaviour disorders (RBD) [31]. However, LC degeneration is not seen in all patients with PD, but more common in patients with severe dementia [32], which is shown in this study, that is, the specificity and sensitivity of ROC of the LC in early stage PD patients are 66.46% and 67.12% whereas in progressive stage are 90.11% and 70.84%, indicating that the LC had a higher specificity in evaluating progressive PD [33]. It is known from histopathological evidence that Lewy body in the LC often appear several years earlier than that in the SN in PD [34]. For this reason, *Braak et al.* proposed the famous “Braak’s stages” of PD according to the chronological order of the occurrence of Lewy body in various parts of the brain [35] despite *Burke et al.* and *Jellinger et al.* questioned its authenticity and predictive value [36, 37]. However, it is known that non-motor symptoms (such as sleep disturbance, anxiety and depression, etc.) associated with neuronal loss in the LC tend to affect PD patients more than that dyskinesia does [38]. And almost all PD patients experience mild cognitive impairment, particularly in the early stage [39], which have been demonstrated in the current study. Animal studies have shown that tyrosine hydroxylase immunoreactive terminals and NE levels(rather than DA) in striatum, olfactory bulb and spinal cord of transgenic mice that expressed human α-synuclein A53T mutant were decreasing in an age-dependent manner, indicating that LC was more susceptible to the toxicity of abnormal α-synuclein than the SN [40]. Growing evidence suggests the important role of the LC in the development of non-motor symptoms in early stage of PD and have an advantage over the SN in evaluating the severity of the disease. However, Luppi PH et al. argued that RBD was due to degeneration of glutamatergic neurons in the dorsolateral pontine tegmental nucleus or in the ventral medullary reticular formation, instead of NE neurons in the LC [41]. Therefore, the role of LC-NE pathway in PD can be complex and diverse and how the LC degeneration contributes to different non-motor symptoms require further research in the future.

The use of NM-MRI has already been used in previous research into PD and the methodology is not entirely novel. However, our work provides new evidence in the following areas. Firstly, we examined neuromelanin distribution at different sections of the SN and different sides of the LC, and their associations with both non-motor and motor experiences. Secondly, due to individual variability regarding non-motor symptoms in PD, we also used NMSS which is a more comprehensive measure than that of MMSE, HAMD, HAMA or MoCA. Thirdly, we evaluated the specificity and sensitivity of the diagnostic value of CNR using NM-MRI in both the early and progressive stages of patients with PD and provided new evidence to compensatory mechanisms in the progression of PD including the LC may compensate for the loss of DA by producing NE. It should be also noted that there are limitations in the current study. Firstly, this is only a cross-sectional study which limits the understanding of the role of NM in disease progression in PD. Secondly, the relatively small number of participants during the restricted COVID period limits the generalisation of our findings. Longitudinal studies with a larger sample size are needed to confirm these findings. Thirdly, the uncontrollable abnormal involuntary movements may cause artifacts and degrade image quality despite an optimised imaging protocol adopted.

## Conclusions

This case control study reveals asymmetrical distribution of neuromelanin and bilateral neuromelanin loss in both SN and LC in patients with PD. The study also reveals that neuromelanin loss was more predominant in the LC than that in the SN in early stage of PD. This highlights that signal changes of the LC detected by NM-MRI combined with psychometric measures may offer an effective biomarker for PD progression. Future longitudinal studies are warranted to validate these findings and characterize how neuromelanin loss in the LC evolves at different stages of PD and to develop multimodal approaches combining NM-MRI imaging with positron emission tomography imaging to understand pathophysiology in PD, that could lead to novel neuropharmacological intervention targets.

## Data Availability

All data reported in this manuscript will be made available from the corresponding author (Professor Teng Chen (email: chenteng@sdu.edu.cn) on reasonable request.
